# Perceptions About Innovative and Traditional Learning Spaces: Teachers and Students in New Zealand Primary Schools

**DOI:** 10.1007/s40841-023-00280-9

**Published:** 2023-03-27

**Authors:** Jo Fletcher, John Everatt, Yogeetha Devi Bala Subramaniam, Ting Ma

**Affiliations:** Faculty of Education, Te Kaupeka Ako, University of Canterbury, Te Whare Wananga o Waitaha, Christchurch, New Zealand

**Keywords:** Teaching spaces, Innovative learning environments, Reading, Primary school students

## Abstract

In New Zealand, the architectural design of schools and the spaces where children learn are being innovated to allow for more opportunities for teachers and students to work collaboratively. However, there is a dearth of research that has investigated both teachers’ and students’ perceptions of the learning spaces. Little attention has been paid to Asian students, who may perceive learning quite differently from their English-only speaking counterparts. This article compares the perceptions of teachers with students, highlighting the group from Asian backgrounds in both innovative with traditional learning spaces. Fourteen Year 5 and 6 primary teachers from traditional and innovative learning environments were interviewed. Additionally, a questionnaire was given to 150 Year 5 and 6 students. The study found that although many of the teachers perceived challenges with noise and distraction in innovative learning environments, this was not as evident in the responses from the students, particularly the Asian students.

## Introduction

An innovative learning environment (ILE) is an ecosystem of people, pedagogical practices, and physical environments (Ministry of Education, 2023). Originating in projects led by OECD’s Centre for Educational Research and Innovation, ILEs are characterised as those that are learner-centred, social and collaborative, highly attuned to learners’ motivations and emotions, and acutely sensitive to individual differences, and those that have demanding learning tasks, use formative assessment, and promotes horizontal connectedness across activities and subjects, in and out of school (OECD, 2013, pp. 177–178).

In New Zealand, innovative learning environments are evident in (a) schools newly built, (b) schools partially rebuilt with the addition of new innovative learning blocks, and (c) existing traditional school buildings converted internally to accommodate innovative learning environments. Teachers in these spaces typically come together to work with a large group of 45–180 students from similar or combined year groups (Fletcher & Everatt, 2021). This could be challenging for teachers to adjust their pedagogical practices to meet a variety of learner needs (Blackmore et al., 2011; Mackey et al., 2017). The challenges centre on collegiality and collaboration among teachers, altering mindsets to embrace the pedagogical shift, and a lack of professional development that prepares teachers to cope with the change.

However, the innovative learning spaces ideally give teachers the opportunity, flexibility, and technology support they need to work collaboratively with one another and the students to bring in interventions that continuously help improve teaching and learning practice (Nicoll, 2016). Osborne (2013) similarly points out that teachers in innovative learning environments can use the flexibility of space and access to the variety of resources these environments afford to enhance and extend the repertoire of pedagogies they can use to suit educational needs. Regarding pedagogical variety, Fisher (2005) and Nair (2014) suggest that innovative learning environments can accommodate a range of teaching styles, from teacher-centred to experiential and inquiry-based learning.

Likewise, a survey of 822 school principals and leaders in Australia and New Zealand conducted by Imms et al. (2017) found that in schools with a relatively high prevalence of open-plan spaces, teachers would be more involved in all aspects of teaching, and students would be more active and interested in learning compared to those with a relatively high prevalence of traditional spaces. Positive student engagement, where students are more involved and interested in learning and feel more connected to their learning environments (Axelson & Flick, 2011), is facilitative of knowledge retention and will increase academic achievement, positive behaviour, and a sense of belonging (Taylor & Parsons, 2011).

Studies have examined teachers’ and principals’ perceptions of innovative learning environments and their efforts to adapt to this environment (Alterator & Deed, 2013; Carvalho et al., 2020; Smardon et al., 2015), but few have investigated students’ perceptions and experiences to see whether the challenges and opportunities of ILEs perceived by teachers and school leaders have indeed resonated with the students. Further, we cannot take a “one size fit all” approach, as nearly 50% of the school children come from multicultural backgrounds (Ministry of Education, 2022). To create a learning space that caters to diverse learner backgrounds, Asian students deserve attention as their cultures tend to contrast distinctly with mainstream European cultures, and failure to engage this group culturally in the ILEs risks positive learning outcomes. It is crucial for learning environments to support a variety of learning styles, cater to individual learners and develop strong pedagogical practices that embrace more flexible use of classroom spaces (Weaver, 2006).

## Literature Review

Studies have shown, from a pedagogical perspective, the challenges teachers face to transit into innovative learning environments (e.g., teacher collaboration) (Everatt et al., 2019; Mackey et al., 2017). The issue of noise, from a design perspective, has also raised concerns over its negative impact on teaching and learning experiences (Everatt et al., 2011; Wall, 2016). Architecturally larger in size, innovative learning environments with poor classroom acoustics can lead to noise that is difficult to mitigate. In a noisy learning environment, students will probably misinterpret teachers’ instructions, while teachers will have difficulty managing classroom behaviour (Wall, 2016). Several studies have identified the detrimental effect that the level of noise can have on learning among younger children (see, for example, Klatte et al., 2013; World Health Organization, 2009). At times, noise may be influenced by aspects of classroom designs beyond the control of teachers (Maxwell & Evans, 2000). For students, constant moderate-level exposure to noise can interfere with classroom activities, impair their ability to perform complex tasks and lead to physical and psychological stress (World Health Organization, 2009). In regard to the influence of noise on language acquisition, Hazan and Barrett (2000) found that children in noisy environments had difficulty using stored phonological knowledge, such as individual phonemes, to restructure degraded speech. However, Gordon-Salant (1985) found that high levels of noise put all phonemes at risk and disrupted the listeners’ auditory discrimination.

Researchers found students were easily distracted and lost their concentration, especially during active listening, if their classroom environment was noisy (Everatt et al., 2019; Gumenyuk et al., 2004). Links between chronic noise exposure and reading acumen were identified in a study by Evans and Maxwell (1997). Their findings showed that children in noisy schools had poorer reading skills than children in quieter schools. The children from the noisy schools were unable to distinguish specific sounds because these were masked by other competing noises. Their speech perception was relatively poor, and that, in turn, affected their reading ability. Similarly, Everatt et al. (2011) found that students in open-plan classrooms are more likely to be distracted by the noise in them and to exhibit off-task behaviours if they are young and/or struggling learners. Generally, younger listeners have been found to perform more poorly in noisy environments, as their ability to listen more effectively under noisy conditions develops in their adolescent years (Nelson & Soli, 2000). Students from an English-as-a-second-language background, as indicated by Nelson and Soli (2000), are likely to have difficulty understanding spoken English in noisy conditions, affecting their ability to listen to the verbal sound of words and connect them to written words and thus reading skills. Likewise, Everatt et al. (2019) asked principals and deputy principals about their perceptions of student achievement in innovative learning environments by using interviews and an online survey. Several respondents indicated that noise in these environments tends to disfavour low-progress learners.

Despite work such as Wall’s (2016), there is a lack of literature examining the effects of the noise level on learning in New Zealand classrooms, especially from students’ perspectives. Further, for students whose first language is not English, challenges they may encounter in the innovative learning environments are more than architectural; they can be primarily cultural. In New Zealand, almost 50% of students identified as non-European Pākehā (Ministry of Education, 2022). This group of students bring to their school-based learning prior knowledge and experiences that arise from their diverse cultures and languages. Effective teachers recognise this diversity and help students build on their existing cultural knowledge in order to engage these learners (Ministry of Education, 2016). Creating new spaces or utilising existing ones that cater to students from various cultures and with diverse learning needs and styles is crucial, as this can potentially realise positive student engagement (Jankowska & Atlay, 2008).

In 2022, Asian students accounted for nearly 14% of all enrolled school children and more than half of non-European Pākehā (Ministry of Education, 2022). Few studies in New Zealand have examined the influence of learning environments on this special group from their perspectives.

In general, Asian children are generally taught to respond to parents, authority, schools, and teachers in ways that differ from what children born and raised in the Western world are taught (Dixon, 2005). In Asian cultures, conserving knowledge is more important than constructing knowledge in learning situations, with Asian students preferring to read widely and trust expert knowledge. In contrast, Western students prefer to question knowledge and form their own opinions (Dixon, 2005).

Asian cultures value collectivism, avoid uncertainty, and believe in power distance (Hofstede, 2011). According to Loh and Teo (2017), power distance plays the most important role in the classroom for Asian students. Power distributed unequally in a society is based on hierarchical order, which helps explain why Asian students who are predominately from higher power distance backgrounds highly respect and value teachers and see them as holding power in classrooms. Further, because Asian cultures are predominantly cultures that favour the collective over the individual, Asian students tend to value group harmony and conform to group norms—values and behaviours often at variance with Western systems of education, which typically encourage active learning through active participation (Hofstede, 2011; Loh & Teo, 2017).

For teachers, the ability to teach in multicultural contexts, to take into account the cultural practices, norms, preferences, and language learning needs of their students, can be a critical determinant of the effectiveness of the teaching and learning within learning environments (Tagg, 2015). It is thus critical to examine teachers’ practices in innovative learning environments and whether there are any adjustments made to cater to Asian students.

In summary, the study aims to examine both teachers’ and students’ perceptions of innovative vs traditional environments, with a focus on the challenges and opportunities they encounter during learning and teaching. Furthermore, the study specifically focuses on a group of students from Asian backgrounds to see whether culture impacts students’ perceptions of the learning environments and teachers’ adjustment of pedagogical practices.

## Methods

The research comprised two phases: a qualitative phase that involved interviews with Year 5 and 6 teachers and a quantitative phase that involved a questionnaire distributed to students in Year 5 and 6 within most of the same New Zealand primary schools as the teachers (teachers in one traditional classroom school in which student data were collected did not volunteer to take part in the interview sessions due to concerns about the pressure of time during the early stages of the Covid-19 pandemic). The qualitative data provided themes on which to describe the views of teachers about innovative learning environments and contrast with views about traditional single teacher classrooms. The student questionnaire data then provided a contrast with the teachers’ views. Schools were selected based on New Zealand data indicating that they were likely to contain a large number of students with English as an additional language. This would increase the chances of accessing a reasonable number of Asian background students in the sample. All schools in the research volunteered to take part in the research based on principal informed consent. Teachers and students were also involved in informed consent procedures as part of volunteering to take part.

### Participants

A total of 14 Year 5 and 6 teachers volunteered to take part in the semi-structured interviews. They were from primary schools in and around one of the larger cities in New Zealand: three schools with traditional classrooms, two schools with innovative learning environments that were refurbished from traditional classrooms, and two schools with innovative spaces that were purposely built. Eight teachers were teaching in innovative learning environments, and the other six were in traditional single teacher classrooms. All were female, and their teaching experience ranged from 2 to 39 years. The average time spent in innovative learning environments by the teachers who, at the time of the study, were teaching in an innovative classroom was over 4 years. The teachers had practised for a minimum of 2 years in the schools in which the research was undertaken, which allowed them some familiarity with these teaching environments in order to discuss in detail their pedagogical practices and challenges encountered.

In addition to the teachers, 150 students from four schools with traditional classrooms (*N* = 69) and four schools with innovative learning environments (*N* = 81) participated in the questionnaire phase of the research. Of the 150 students, 71 were from Asian backgrounds, and 79 were from English-only-speaking backgrounds (European Pākehā). As well as English, the languages spoken by the Asian students at home comprised Chinese (mostly Mandarin), Japanese, Hindi, Kannada, Tamil, Urdu, Malayalam, Sinhala, Malay, Thai, Korean, Vietnamese, Bisaya and Tagalog. The students had lived in New Zealand for a varied amount of time, but on average, 96.49 months.

### Instruments

Semi-structured interviews were conducted with the teachers in school terms 3 and 4, which would have allowed teacher–student rapport building during the previous terms (Cohen et al., 2018). Teachers were asked to identify the pros and cons of teaching in the classrooms of their present school, particularly aspects of the learning environment that enhanced their teaching or that presented challenges. Additional questions were also asked about teaching students from an Asian background. The questions for the interview were sent to the teachers 3 days before the interview to allow time for the teachers to reflect beforehand. All interviews were audio-recorded, and pseudonyms were used in transcribing the recordings.

The example of teaching reading was used in the interviews and questionnaires to give a specific context for perceptions about teaching in different types of classrooms. Reading is an integral part of literacy instruction for Years 4–8 students and is key to the acquisition of the English language and the development of competencies of “thinking and using language, symbols, and texts” (Ministry of Education, 2009, p. 5). Reading achievement has been a focus of heated discussion, as NZ primary school students showed weakened performance in the Progress in International Reading Literacy Study (PIRLS) 2016, compared to 2011 (Chamberlain, 2019). Moreover, the percentage of changes in reading education practices in New Zealand has been much lower than the OECD average between 2006 and 2016, according to the OECD’s report on measuring innovation in primary and secondary education (Vincent-Lancrin et al., 2019). The context of teaching reading was thus deemed an appropriate example to use. It would help teachers frame the opportunities and challenges they faced while teaching in traditional or innovative learning environments and the children to reflect on learning experiences, which was useful for consistent comparisons across teacher and student responses.

A questionnaire was developed to elicit students’ perceptions of innovative and traditional learning environments. There were 32 items, with responses given on a five-point Likert scale, indicating frequency from Never, Rarely, and Sometimes to Often and Always. Items formed five scales: Attitudes to Reading, Teacher Support, Equity, Noise, and Conduciveness. Seven out of ten questions on Attitudes to Reading were taken from the *Revised Learning Process Questionnaire* by Kember et al. (2004). The remaining three questions on attitudes were developed by the authors. There were eight items on Teacher Support and five on Equity. These were adapted for the New Zealand and reading-example context from Charalampous and Kokkinos (2017)and Fraser et al. (1996, August 31). The five items on Noise and the four on Conduciveness were developed by the authors. The questionnaire was piloted among Asian and Non-Asian students to ensure the langue was clear and easy to understand. Based on students’ feedback, some questions were reordered and reworded to ensure clarity and avoid misunderstanding.

All questionnaire items related to the example of reading learning, class, or practice in order to give the students focus on which to answer the items—more general statements may have been harder for the children to answer in this questionnaire format. Reading was chosen as the focus, as all students would be participating in reading time, given that it is a core skill and it is an area where noise and a distractive environment may lead to negative consequences. However, differing attitudes to reading may lead to more positive or negative responses, so an additional set of items asked the participants to indicate their Attitudes to Reading. These items included ‘I like reading’, ‘I try to use what I have learned in my reading class to help me in my other subjects’ and ‘I do only what the teacher wants me to do in my reading class and nothing extra’ (this latter item was reverse coded so that a high score represented more positive attitudes). The Teacher Support items included ‘My teacher supports me during reading time in class’ and ‘My teacher has helped me to improve in my reading’. For Equity, items included ‘I get the same amount of help as the other students from the teacher during reading time’ and ‘I get the same amount of encouragement from the teacher as other students do during reading time in class’. The Noise items included ‘The class environment is noisy during reading time’ (this item was reverse coded so that higher scores represented more positive perspectives on learning, and given that too much noise would be considered negative) and ‘I can clearly hear what the teacher is saying during reading time’. The Conduciveness items included ‘I like my classroom environment during reading lessons’ and ‘I like how the reading activities are carried out in the class I am in’.

### Data Analysis

Teachers’ interviews were transcribed and thematically analysed. Thematic analysis offers flexibility during data analysis because it is “not wedded to any pre-existing theoretical framework” (Braun & Clarke, 2006, p. 81). However, the researchers’ own positioning and assumptions may arguably be influential. Thus, we identify ourselves as academics who have an interest in how different learning spaces may impact learning and teaching. Data were first sorted according to the types of learning environments (innovative versus traditional). The analysis steps proposed by Clarke and Braun (2017) were used to capture patterns within the data sets. Next, codes were developed specifically for the key points in the interview extracts (e.g., challenges of teaching and behaviour management). After assembling the coded information, which was in the form of extracts from the interviews under the themes, each extract was checked to see whether it had been correctly coded and aligned with the themes.

Students’ questionnaire data were processed by the Statistical Package for Social Sciences (SPSS, Version 28.0). Answers for each item were coded from 1 to 5—with 5 being a more positive answer. Scores for each of the five scales were produced by averaging the children’s responses to the items on each scale. These scale scores were then analysed using analyses of variance (ANOVA) to identify differences in students’ perception between Asian versus English-only-speaking groups and between innovative versus traditional environments.

## Results

### Teachers’ Perceptions

The emerging themes developed from analysing teachers’ interviews focused on the challenges of potentially noisy environments and related aspects of conduciveness of classroom spaces for different pedagogical practices, as well as the importance of teacher support and maintaining equity in learning for all students, including those from an Asian background. The themes are discussed below, with examples taken from the individual teacher’s responses (note that pseudonyms are used throughout).

### Challenges of Noise

Noise in teaching has long been identified as having a negative effect on students’ attention, speech perception, and educational outcomes (Everatt et al., 2011; Nelson et al., 2005; Wall, 2016). In innovative learning environments, dealing with noise has been challenging for student teachers, teachers, and principals (see, for example, Everatt et al., 2011, 2019; Fletcher & Everatt, 2021). Teachers from the current study also expressed their concern over this issue.

The noise is really loud and the fact that your kids, if you’re not keeping an eye on them, they’ll get up and they’ll move around, or be talking to someone else, or they won’t be doing what they’re meant to be doing. It just makes it a lot harder to control because of the big space, with more noise. (Wendy, *Innovative Learning Environment*)

Teachers’ comments below also suggested that noise has posed great difficulty for Asian students to pick up new words and interpret classroom instructions.

Just the noise, you know, because there are so many extra kids. Trying to pick up new words that they’ve never learned before. (Jill, *Innovative Learning Environment*)

It’s all about visuals…It helps them because it gives them a sense of structure. I think for them [Asian students] especially, the language barrier can make it a little bit difficult for them to figure out where they need to be or what they need to do. So having the wall up there with their faces for, like, Monday, Tuesday, Wednesday, they know exactly where they need to be at that time. (Sharon, *Innovative Learning Environment*)

The above observations align with studies on the detrimental effect of noise on learning. Young learners (especially those with learning difficulties) tend to be distracted by the noise in open-plan classrooms and thus have difficulty performing classroom activities and tasks (Everatt et al., 2011). For English language learners, noise may impair their ability to connect pronunciation with spelling and writing (Nelson & Soli, 2000).

However, observations by teachers in the current study form an interesting contrast with the 222 primary school teachers who participated in a New Zealand national survey on their experiences of ILE and traditional learning environments (Carvalho et al., 2020). In the survey, most primary school teachers (92%) could hear students clearly and did not seem to be disturbed by the noise in their classroom. Of these teachers, 15% were from traditional learning environments, 31% from innovative ones, and 51% from learning environments that were in the process of transiting to innovative ones.

However, teachers from innovative learning environments felt that the negative impact of noise could be alleviated by effectively managing students’ behaviour and using visuals for teaching instructions.

The biggest challenge would be if you probably didn’t have your behaviour management, or your noise level is up, that you couldn’t actually keep that nice quiet time to actually have quality conversations with the groups that you’re actually working with. I mean, if you have really good routines and really good behaviour management, then the kids just follow and it’s easy. (Jill, *Innovative Learning Environment*)

This highlights the importance of aligning pedagogical practices with the affordances of physical learning environments (French et al., 2020), where in the case of innovative learning environments, it is common to accommodate a large number of students and multiple teaching activities because of the large and flexible space.

In contrast, this study found that the teachers perceived that noise levels tend to be lower and easier to manage in traditional single teacher classrooms. Teachers’ comments below suggested several advantages of quieter environments than those provided by an innovative learning space. These included students being able to focus and capture accurate pronunciation and teachers being able to identify individual learning needs because of smaller class sizes.

So, they [children] are able to hear the words, or the way things are articulated or pronounced, perhaps more easily than if there was the surround-sound of other children’s voices. (Tracy, *Traditional Single Teacher Classroom*)

You just have fewer distractions. You can just concentrate on those children in your classroom. You’re not having to worry about comings and goings of children, other things happening in the wider environment. (Emily, *Traditional Single Teacher Classroom*)

It appeared that fewer distractions, a smaller physical space, and lower noise levels were not only seen as advantageous to the learners but equally applicable to the teacher’s ability to concentrate and teach in a conducive teaching space.

### Conduciveness of Classroom Spaces

Connected to ideas related to noise and distraction in innovative learning environments, there was also discussion of how these spaces, designed to promote team teaching (Kedian & West-Burham, 2017; Starkey & Wood, 2021), were ineffectively used and explored. For instance, differences in teaching styles and classroom behaviour management were perceived as factors hampering teacher collaboration, as shown in the comments below.

I think in some cases, if you are with certain other teachers who may not have good behaviour management, collaboration becomes difficult. (Karen, *Innovative Learning Environment*)

Teachers will have their own ways of working and that doesn’t always work for the other teachers around them. (Debra, *Innovative Learning Environment*)

The differing teaching styles and expectations of the team of teachers within innovative learning environments were problematic for some teachers. Indeed, the successful implementation of team teaching demands great adaptability and flexibility from teachers (Alterator & Deed, 2013) and their willingness to create a shared vision and collaborative practices (Carvalho et al., 2020). However, in traditional classrooms, although teachers could choose teaching styles, they had difficulty meeting all individual students’ learning needs effectively because of limited time. They also experienced pressure because they were the only teacher in the class without help from another one, and students had no choice but to accept the teacher they were assigned to.

When you look at the amount of time a teacher has with each child and you’ve got thirty children in the class, you always want more time. And I don’t think we could do a lot different because you’ve sort of got to do your best to spread your time evenly across the children, so I couldn’t do anything different. Unless I split myself in two. I do miss sometimes working with another teacher; you can bounce ideas off each other. (Kate, *Traditional Single Teacher Classroom*)

On the other hand, the advantages of teaching in innovative learning environments offered flexibility and collegiality, which some teachers perceived was beneficial for catering to a wide range of learning needs. This enabled one teacher to have quality learning with students whilst the other teacher(s) focused on the remaining of the class.

### Teacher Support

In high power-distance societies such as Singapore, Malaysia, Hong Kong, China, South Korea, Indonesia, Vietnam, Thailand and the Philippines, the notion of ready access to teacher experts is deeply embedded in the cultures (Loh & Teo, 2017). This is characterised frequently by students showing respect and not questioning the teacher’s authority.

As shown in the comments below, in innovative learning environments, having more than one teacher in their classroom meant teachers could rely on one another to assist Asian students who required additional help with their English-language reading. This also meant that the primary teachers could continue with reading instructions without having to pause to assist students needing extra guidance.

It’s about having that time. If you were the only one in the classroom with your thirty kids, then you can’t be pulled away that quickly. Because there are a couple of teachers available, you can call someone out for five, ten minutes and have the conversations or the teaching or just to set them up and then slot them back in. (Jill, *Innovative Learning Environment*)

However, the teachers from the traditional schools spoke about the close teacher–student relationships because of the smaller number of students in this environment, which enabled prompt responses to students’ needs.

I know every single child in my class really well and I know what they enjoy. I know them as a learner; I know about them. I have that relationship, and if something’s not going well, I can pick up on that and help support them with possibly doing something different at the time if they need to or having a bit of a time out. So, I think that’s probably one of the things; definitely the relationships with the children, I think, is key to learning. (Jane, *Traditional Single Teacher Classroom*)

As Loh and Teo (2017) suggest, ready access to the teacher not only gives Asian students an opportunity to clarify and to learn but also confidence that they are learning from experts. For that reason, it may be perceived to be more conducive to learning styles for Asian students to have only one teacher whom they see as their specific teacher rather than a team of teachers, as can be the situation in innovative learning environments. Nevertheless, in innovative learning environments, one of the teachers is often designated to a large group of children, and the students have access to one specific teacher and at the same time, are able to interact with the team of teachers. Arguably, in either innovative learning environments or traditional single teacher classrooms, Asian students should have ready access to teachers who are able to support their learning.

### Cultural Equity

Responses from teachers in innovative learning environments suggested that the structure of the learning environment made no difference to how they taught reading to Asian students. Overall, they were teaching reading to these students the same way they were teaching their English-only-speaking students.

They [Asian students] end up coming out at different times of the day for extra support for mainly reading and comprehension and things like that; otherwise, we still just do it as normal with them in our groups. I’ve got two that I have to keep an eye on because they don’t fully comprehend exactly what’s going on; I just check in with them. But I haven’t changed my programme as such. (Wendy, *Innovative Learning Environment*)

Teachers in the traditional schools appeared to have a better grasp of the diverse cultural backgrounds of the Asian students in their classrooms and the need to tailor their reading programmes to meet the needs of those students. For instance, there was awareness of the differences in the wider Asian community and the need to cater to these differences.

Asians, you know, it’s quite a big, huge geographical label ... It’s too broad. I mean, we used to have children here who have come from Korea. Now they were very different, and you know, now we’ve got a lot of Filipino children, and they are very different to the Korean children. It’s hard to have to generalise. You have to cater to individual needs. (Emily, *Traditional Single Teacher Classroom*)

This may be because the teachers in the traditional schools had a smaller number of students overall in their classes, which made it easier to develop close teacher–student relationships and gain better knowledge of their cultural backgrounds. Nevertheless, the student-to-teacher ratio in state schools is set, regardless of whether it is an innovative learning environment or a traditional single teacher classroom.

However, teachers from innovative learning environments, although lacking in certain practices that cater to Asian cultural backgrounds, expressed their willingness to support their Asian students by incorporating reading resources related to students’ cultural backgrounds and experiences.

And sometimes, you know, some of the Asian children coming through, with the materials that we’ve got, they sort of go, “What is this all about?” Because they don’t know what it’s about. So that can be quite challenging. So if I have, like, books from their home countries or about their home countries, but in English, I don’t know how that could be done. But that would definitely be an advantage for them. (Debra, *Innovative Learning Environment Tw*o)

This aligns with the literature about the cultural influence on students’ learning outcomes. Gay (2018) emphasises that culturally responsive practice should draw on students’ personal and cultural strengths in order to create learning experiences meaningful to them.

## Students’ Perceptions

The interview data from the teachers provided one lens, but the views of the 150 students collected by a questionnaire were also sought in this research. Students’ responses to the 32 questions on five scales were coded from 1 to 5, with 5 being a more positive answer. Table 1 presents the mean scores (with standard deviations in brackets) of the four groups of students on the five scales. The results of the two-way analyses of variance for each scale are also presented in Table 1.

**Table 1:** Students’ perception of learning spaces

	Asian background (*N* = 71)		English-only-speakers (*N* = 79)		ANOVA^a^
	Traditional (*N* = 28)	Innovative (*N* = 43)	Traditional (*N* = 41)	Innovative (*N* = 38)	
Attitudes to reading	3.49 (0.46)	3.63 (0.51)	3.58 (0.50)	3.30 (0.70)	*F*(1, 149) = 1.74, *p* = .19 *F*(1, 149) = 0.56, *p* = .46 *F*(1, 149) = 5.22, *p* = .02*
Teacher support	3.25 (0.57)	3.42 (0.57)	3.63 (0.57)	3.26 (0.66)	*F*(1, 149) = 1.09, *p* = .30 *F*(1, 149) = 0.87, p = .35 *F*(1, 149) = 7.58, *p* = .01*
Equity	3.61 (0.84)	3.69 (0.79)	3.92 (0.75)	3.70 (0.97)	*F*(1, 149) = 1.29, *p* = .26 *F*(1, 149) = 0.27, *p* = .61 *F*(1, 149) = 1.10, *p* = .30
Noise	3.58 (0.59)	3.40 (0.67)	3.48 (0.57)	3.24 (0.77)	*F*(1, 149) = 1.35, *p* = .25 *F*(1, 149) = 3.71, *p* = .06 *F*(1, 149) = 0.08, *p* = .78
Conduciveness	3.75 (0.66)	3.73 (0.68)	3.82 (0.77)	3.49 (0.75)	*F*(1, 149) = 0.56, *p* = .45 *F*(1, 149) = 2.13, *p* = .15 *F*(1, 149) = 1.73, *p* = .19

*Indicates significant results

^a^ANOVA results are presented in the order of the main effect of student backgrounds, the main effect of learning environments, and the interaction effect between learning environments and student backgrounds

The results indicate two significant interaction effects for Attitudes to Reading and Teacher Support. In both cases, the main difference between innovative and traditional conditions is for the English-only students, where those in the innovative classrooms responded with less positive Attitudes to Reading (*M* = 3.30) and lower scores for Teacher Support (*M* = 3.26). In contrast, the Asian background students in the innovative learning classrooms were more positive with higher scores on these two scales, Attitudes to Reading and Teacher Support, than their Asian peers in traditional classrooms.

Indeed, for all measures, there are only small differences between the average responses of the Asian background students in the two classroom environments. The bigger differences are for the English-only students, with those in the innovative classrooms consistently showing a less positive attitude by scoring lower than their English-only peers in the traditional classrooms. The larger effects for these English-only students are for Attitudes to Reading and Teacher Support (Cohen’s *d* = 0.46 and 0.60) but smaller for Equity (Cohen’s *d* = 0.25), Noise (Cohen’s *d* = 0.35), and Conduciveness (Cohen’s *d* = 0.43).

The results indicate that the impact of innovative learning environments on students’ perceptions was less evident for Asian background students than English-only students, who were more positive overall about learning in traditional single teacher classrooms. This may be because Asian cultures tend to value group harmony and conformity, which are values and behaviours that contrast with Western values, which tend to foster active learning and participation (Hofstede, 2011; Loh & Teo, 2017). Arguably, Asian students may be more aligned then to succeed in either type of learning environment. However, caution with generalising these results is needed due to the sample size.

The quantitative results seemingly misalign with previous studies that investigated the relationship between the physical learning environments and students’ attitudes towards learning experiences (see, for example, Byers et al., 2014). For instance, Byers et al. (2018) compared Australian secondary school students, regardless of ethnicity, from both types of learning environments and identified positive effects of innovative learning environments on students’ assessment of emotional, behavioural, and cognitive engagement. However, in our study, where English-only speaking students and Asian students were viewed separately, the English-only speaking students exhibited fewer positive attitudes towards learning in innovative learning environments compared to learning in traditional classrooms. Further, the Asian students did not seem to vary significantly in their attitudes in all the five areas (Attitudes to Reading, Teacher Support, Equity, Noise, and Conduciveness) across the two different types of environments. This misalignment seems to indicate that Asian students are less influenced by classroom design than their English-speaking peers.

## Discussion

Byers et al. (2018) propose that being in an innovative learning environment alone does not guarantee a positive pedagogical mentality and change. Instead, it is the teachers, as capable mediators between the affordances of innovative learning environments and suitable pedagogical practices, who are the catalyst to change. What is of interest in our study is that the comparison of quantitative and qualitative results shows several discrepancies between students’ and teachers’ perceptions of noise, conduciveness, and teacher support related to learning environments.

Interviews with the 14 teachers found that many had a significant concern over how noise in innovative learning environments may distract students from learning and reading. For example, the level of the noise may mean the subtle pronunciation of words is not clearly heard and prevent Asian students, for whom English is a second language, from acquiring new vocabulary and understanding classroom instructions. However, statistical analysis of students’ questionnaire data showed that both Asian and English-only speaking students did not consider innovative learning environments to be significantly noisier than traditional classrooms. This could be because some pedagogical practices recommended by the teachers appeared to be effective in regulating the negative effect of noise in innovative learning environments. These include, but are not limited to, working with and supporting teaching colleagues, managing students’ behaviour, and using visuals for instructions. That noise was not perceived to be interfering, especially with the Asian students, could also be because the teachers were aware of the severity of noise and thus deliberately acted to lessen such a negative impact, including retreating to a small area free from either teacher or student interactions. Students may also have adapted themselves well to the arrangement of their innovative learning environments by developing capacities to effectively interact with course materials and resources as well as with peers to construct knowledge. Indeed, learning is not only about clearly hearing one voice (Carvalho et al., 2020). Arguably, students may be more adaptable to noise levels than teachers, who are leading teaching and managing complex learning and teaching plus dealing with a range of interrelationships, moment by moment, throughout the teaching day.

Students appeared to consider both types of learning environments equally conducive to learning, while the teachers in innovative learning environments identified challenges of noise and distraction that could impede learning and differences in teaching styles and student behaviour management that could impede teacher collaboration. Indeed, team teaching requires collaboration in many areas, such as timetabling, course planning, and assessment discussion; teachers of a team are interdependent on each other (Blackmore et al., 2011). The teachers’ comments, to some extent, indicate teachers’ unpreparedness to use innovative learning environments effectively (Johnston, 2022) and thus calls for investment in teaching practices through professional development (French et al., 2020). Teachers in traditional settings also identified the pressure of working alone, not having immediate collegial teacher support and having to deal with groups of students within a fixed amount of time.

Results showed that the teachers in the innovative learning environments did not seem to make substantial changes in programmes that catered to the learning needs of Asian students. However, Asian students did not perceive themselves to be at a distinct disadvantage in innovative learning environments, as they reported a positive perception of available teacher support. The English-only-speaking students, on the other hand, reported in the questionnaire data considerably less teacher support in innovative learning environments than their peers in the traditional classrooms.

## Conclusions

This study’s findings showed that teachers from traditional single teacher classrooms and innovative learning environments reported the number of distractions and the level of noise in innovative learning environments could hamper learning, especially for Asian students. Yet, the results from the quantitative data analyses of students’ perceptions countered the concerns the teachers had around noise level and distractions, with overall, the students reasonably positive about noise not being an issue for their learning. However, the quantitative data were reported as a mean score, and possibly some of the outlying lower scores, which indicate that noise was an issue, may have been from the Asian students. This is an intriguing finding, which requires further research investigation to uncover if the Asian students in learning are more or less positive about noise and distraction in the two different learning environments.

On the other hand, with the complexities within teaching environments, what seems apparent from the findings is that some teachers enjoyed working collaboratively in innovative learning environments and had developed strategies and systems to address the issues around maintaining a conducive noise level and lowering the number of distractions. Though for other teachers, their relationships and differing expectations around noise and distractions, plus other opposing teaching strategies, appeared to lead to teacher frustration and most likely a less conducive learning environment for the students to learn within.

A salient finding was that the Asian students were more positive overall in their attitudes, either in innovative learning environments or single teacher classrooms, than their English-only-speaking peers. This raises the probability that Asian students could be more adaptable and resilient and have values that support learning with a determination to be successful in their schooling, regardless of the type of learning environment. Further research is needed, preferably through student interviews and classroom observation, to uncover why there are differences in the attitudes of the two groups of students towards learning environments.
